# Self‐Powered UV Dual‐Band Photodetector Based on Cs_3_BiCl_6_/GaN Heterojunction for Logical Operation and Encrypted Photo‐Communication

**DOI:** 10.1002/advs.202503498

**Published:** 2025-05-11

**Authors:** Jingli Ma, Fei Zhang, Yakun Xing, Huifang Jiang, Yongtao Tian, Huifang Ji, Xu Chen, Di Wu, Longhui Zeng, Xinjian Li, Chongxin Shan, Zhifeng Shi

**Affiliations:** ^1^ Key Laboratory of Materials Physics of Ministry of Education School of Physics Zhengzhou University Daxue Road 75 Zhengzhou 450052 China; ^2^ School of Flexible Electronics (SoFE) Henan Institute of Flexible Electronics (HIFE) Henan University 379 Mingli Road Zhengzhou 450046 China; ^3^ Department of Applied Physics KTH Royal Institute of Technology Stockholm S‐10691 Sweden

**Keywords:** Cs_3_BiCl_6_, encrypted photo‐communications, lead‐free perovskites, logical operations, UV dual‐band photodetectors

## Abstract

Dual‐band photodetectors have huge potential for application in secure optical communication, multicolor imaging, and logical operation. However, the majority of currently documented dual‐band photodetectors suffer from high energy consumption and poor photoresponse performance; especially, the dual‐band photodetectors targeted at the UV region have yet not been reported. In this study, for the first time, a self‐powered UV dual‐band photodetector based on Cs_3_BiCl_6_/GaN heterojunction is designed and fabricated through a dual‐source co‐evaporation technique. Experiments and theoretical simulations confirm that the unique dual‐band absorption characteristics of Cs_3_BiCl_6_ and the strong surface‐charge recombination near the Cs_3_BiCl_6_ surface are the primary factors enabling the achievement of UV dual‐band photodetection. Importantly, owing to the well‐matched energy band alignment of Cs_3_BiCl_6_ and GaN, the photodetectors exhibit ultra‐high *I*_on_/*I*_off_ ratio (1 × 10^7^), large specific detectivity (1.23 × 10^12^ Jones), and ultrafast response speed (τ_r_/τ_f_ = 28 µs/190 µs). Finally, utilizing the UV dual‐band characteristics of the fabricated device, the logical operation and encrypted photo‐communication applications are successfully demonstrated. The obtained results suggest that the lead‐free perovskite Cs_3_BiCl_6_ is potentially an attractive candidate for the manufacture of high‐performance UV dual‐band photodetectors that can be employed in advanced encryption technology.

## Graphical abstract

Utilizing the special dual‐band absorption characteristics of Cs_3_BiCl_6_ and its strong surface‐charge recombination behavior, a self‐powered UV dual‐band photodetector based on Cs_3_BiCl_6_/GaN heterojunction is developed. As a result, the photodetector exhibits excellent specific detectivity of 1.23 × 10^12^ Jones and ultrafast response speed of *τ*_r_/*τ*_f_ = 28 µs/190 µs. Further, the photoelectric logical operation and encrypted photo communication are successfully realized.

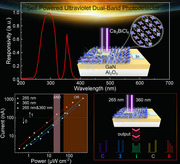

## 1 Introduction

Photodetectors can convert incoming optical signals into electrical signals to realize the transmission of information, providing great possibilities for the future development of the Internet of Things, quantum computers, and intelligent life.^[^ ^1^, ^2^, ^3^ ^]^ Nevertheless, conventional single‐band photodetectors are at risk of signal leakage,^[^ ^4^ ^]^ and broad‐spectrum photodetectors encounter signal interference because they respond to multiple optical wavebands.^[^ ^5^ ^]^ In comparison, the dual‐band photodetector is able to process the signals from two specific optical wavebands. If the eavesdropper does not know the correct modulation wavelength and modulation mode, the information will be erroneously decoded, which aids the encryption of the information.^[^ ^6^ ^]^ Therefore, the dual‐band photodetectors have significant application prospects in encrypted photo‐communication, logical operation, optical guiding, etc.^[^ ^6^, ^7^, ^8^ ^]^

Up to now, there have been several strategies to realize dual‐band photodetection, for instance: 1) connecting two single‐band photodetectors in parallel, but this will increase the manufacturing complexity;^[^ ^9^ ^]^ 2) using the alloy material as the photoactive layer, but the material is prone to phase separation;^[^ ^10^ ^]^ 3) fabricating superlattices and multiple quantum well structures, but it has to strictly control the strain and overcome the lattice mismatch between the semiconductor layers in the epitaxial growth.^[^ ^11^, ^12^, ^13^ ^]^ In comparison, constructing heterojunction by combining properly two materials with different light absorption has proved to be a feasible and effective strategy to achieve dual‐band photoresponse. For instance, Zhao et al. prepared a photoelectric multiplier detector with the structure of ITO/PDIN/P3HT:PC_61_BM/Al and the device achieves dual‐band photoresponse under the bottom illumination/reverse bias and top illumination/forward conditions.^[^ ^14^ ^]^ However, organic photodetectors generally suffer from a compromise in response speed due to inferior carrier mobility.^[^ ^15^ ^]^ Therefore, from the application point of view, it is imperative to develop inorganic photoactive materials with excellent photoelectric properties for manufacturing dual‐band photodetectors.

Recently, metal‐halide perovskites have emerged as a promising candidate for photodetector applications owing to their superior optoelectronic properties including adjustable band gap, large absorption coefficient, high carrier mobility, and so on.^[^ ^16^, ^17^, ^18^, ^19^ ^]^ By constructing heterojunctions with other semiconductors, much progress in dual‐band photodetectors has been witnessed in recent years. For example, Zhang et al. prepared SnO_2_ microwires/CsPbBr_3_ particles complex photodetector, which demonstrates UV–vis dual‐band response and achieves self‐powered operation due to the formation of built‐in electric field.^[^ ^20^ ^]^ Bian et al. demonstrated a self‐powered FAPbI_3_/silicon hybrid structured photodetector, which shows visible and near‐infrared bipolar photoresponse.^[^ ^21^ ^]^ By designing the bulk perovskite heterostructures of (C_4_H_9_NH_3_)_2_PbI_4_/(C_4_H_9_NH_3_)_2_(CH_3_NH_3_)Pb_2_I_7_, Wang et al. achieved a narrow dual‐band photodetector operating at 540 and 610 nm with a high on‐off current ratio of ≈10^3^.^[^ ^22^ ^]^ Despite the inspiring progress that has been made on perovskite‐based dual‐band photodetection, previous studies have mainly focused on visible/infrared‐light detection, and dual‐band photodetectors targeted at the UV region are rarely reported. Moreover, the previous studies always employ lead‐based perovskite as the photoactive layer, but its environmental instability and lead toxicity have become a stumbling block that limits further applications.^[^ ^23^, ^24^, ^25^, ^26^ ^]^ Thus, exploring lead‐free and environmentally stable perovskites to solve the above obstacles is urgent.^[^ ^27^, ^28^, ^29^, ^30^ ^]^ Excitingly, the newly emerging lead‐free perovskite derivative Cs_3_BiCl_6_ has intrinsic dual‐band absorption characteristics, providing potential applications for dual‐band photodetectors.

Following this line of thought, for the first time, a high‐performance self‐powered UV dual‐band photodetector was demonstrated based on the Cs_3_BiCl_6_/GaN heterojunction, in which dense nonporous Cs_3_BiCl_6_ films were prepared by dual‐source co‐evaporation technique. Compared to the solvent method, the dual‐source co‐evaporation technique has an unambiguous advantage in the preparation of Cs_3_BiCl_6_ films owing to its solvent‐free manufacturing, weak dependency on substrates, high controllability, and large‐area fabrication.^[^ ^31^, ^32^ ^]^ By combining the experimental results and light field theoretical simulation, we found that the UV dual‐band photoresponse originates from the special two‐band absorption characteristics of Cs_3_BiCl_6_ and the strong surface‐charge recombination close to the Cs_3_BiCl_6_ surfaces. The device performance is remarkable in terms of a high *I*_on_/*I*_off_ ratio of 1 × 10^7^, large specific detectivity of 1.23 × 10^12^ Jones, ultrafast response speed of *τ*_r_/*τ*_f_ = 28 µs/190 µs, and good switch cycle stability. Finally, we successfully demonstrated the applications of such self‐powered UV dual‐band photodetector in logical operation and encrypted photo‐communication.

## 2 Result and Discussion

**Figure 1:**
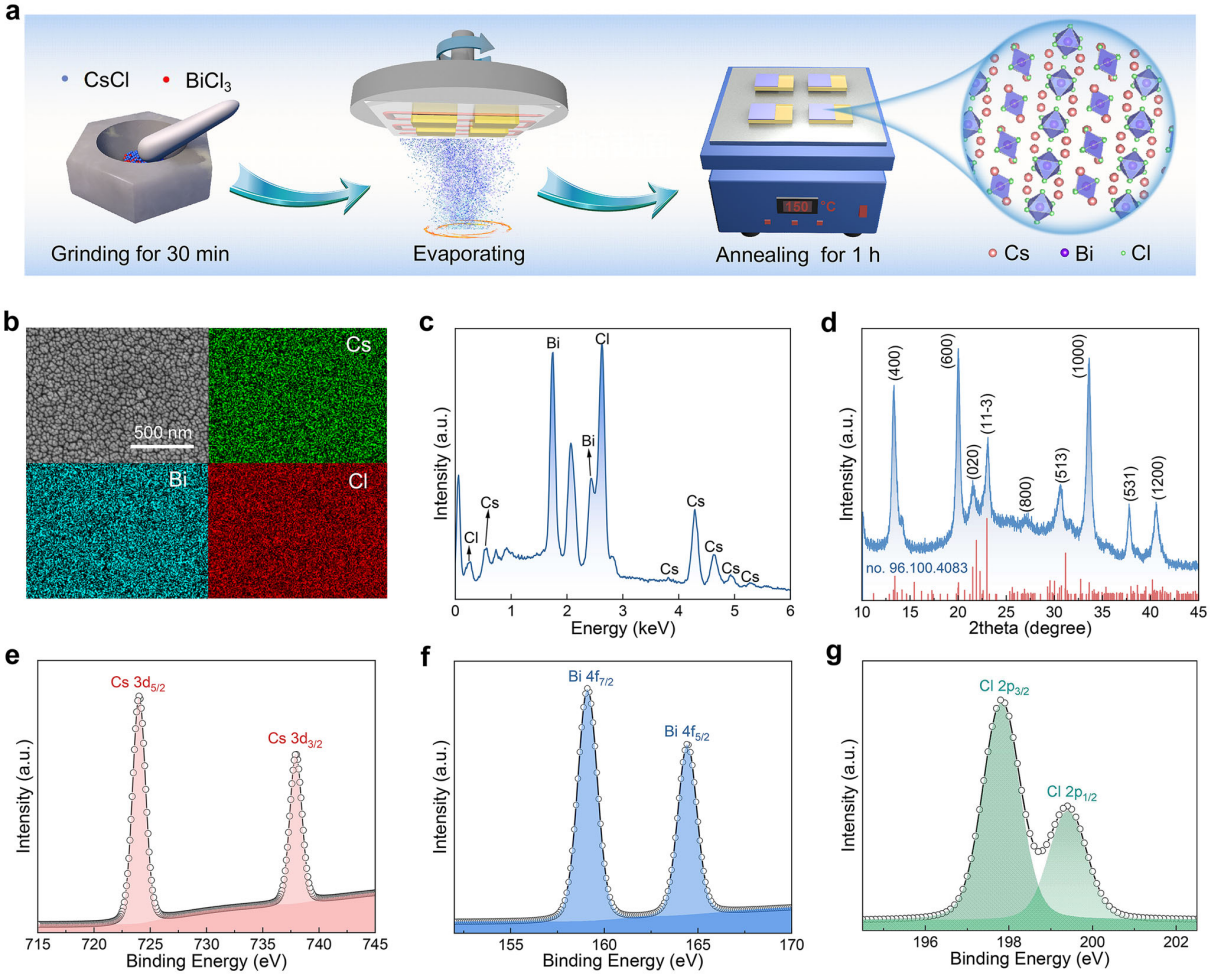
a) Schematic diagram of the preparation process of Cs_3_BiCl_6_ films grown on an *n*‐type GaN template. The inset presents the crystal structure diagram of Cs_3_BiCl_6_. b) Top‐view SEM image of Cs_3_BiCl_6_ films and corresponding elemental mapping images. c) EDS spectra of the Cs_3_BiCl_6_ films. d) XRD patterns of Cs_3_BiCl_6_ films. XPS spectra of Cs_3_BiCl_6_ films for e) Cs 3d, f) Bi 4f, and g) Cl 2p, respectively.

**Figure** **1**a depicts the schematic diagram of the preparation process of Cs_3_BiCl_6_ films grown on the GaN template by dual‐source co‐evaporation technique. Briefly, CsCl and BiCl_3_ with a molar ratio of 3:1 were firstly ground for 30 min in the nitrogen‐filled glove box. The resulting ground powder was then transferred to a molybdenum boat to prepare the Cs_3_BiCl_6_ films through a dual‐source co‐evaporation technique. Finally, the Cs_3_BiCl_6_ films were annealed at 150 °C for 1 h to enhance the crystallinity. Figure 1b displays the scanning electron microscopy (SEM) image of as‐prepared Cs_3_BiCl_6_ films, which presents flat and compact morphology with an average grain size of ≈80 nm. The energy dispersive X‐ray spectroscopy (EDS) elemental mapping results reveal that the Cs, Bi, and Cl elements are uniformly distributed. The EDS spectrum quantitative analysis shown in Figure 1c confirms an atomic ratio of Cs:Bi:Cl as 3:1.1:5.8, close to the stoichiometry of the Cs_3_BiCl_6_ material. Figure 1d displays the typical X‐ray diffraction (XRD) patterns of as‐prepared Cs_3_BiCl_6_ films; all characteristic diffraction peaks well correspond to the standard diffractions of monoclinic Cs_3_BiCl_6_ (space group C2/c, JCPDS no. 96.100.4083), implying the pure‐phase Cs_3_BiCl_6_ films were successfully prepared.

The schematic crystal structure of monoclinic Cs_3_BiCl_6_ was illustrated in the inset of Figure 1a, which is characterized by a 0D electronic structure formed by isolated [BiCl_6_]^3−^ octahedrons and surrounding Cs^+^ ions filled in the space. Figure S1 (Supporting Information) presents the total X‐ray photoelectron spectroscopy (XPS) spectrum of the Cs_3_BiCl_6_ films, in which the signals associated with three elements were detected. Further, the high‐resolution XPS spectra of Cs 3d, Bi 4f, and Cl 2p were analyzed to investigate the chemical bond configurations of three elements, as seen in Figures 1e–g.

**Figure 2:**
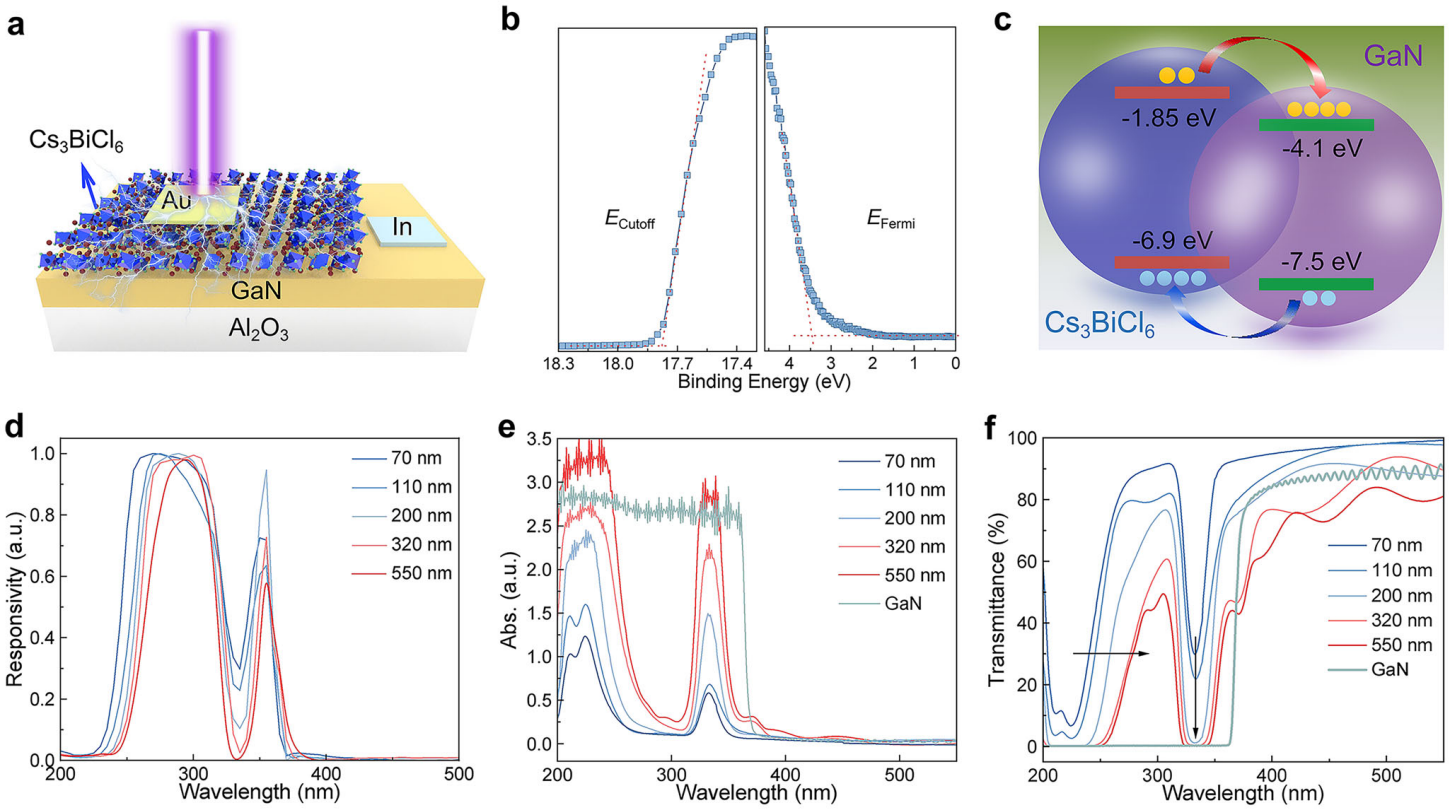
a) Structure diagram of the Cs_3_BiCl_6_/GaN heterojunction photodetector. b) UPS spectra of the as‐prepared Cs_3_BiCl_6_ films. c) Energy band alignment of the Cs_3_BiCl_6_/GaN heterostructure. Yellow circles denote photogenerated electrons, and sky‐blue circles represent photogenerated holes. d) Normalized response spectra of the photodetector with varying thicknesses of the Cs_3_BiCl_6_ layer. e) Absorption spectra and f) transmission spectra of the Cs_3_BiCl_6_ films with varying thicknesses and GaN.

**Figure** **2**a displays the schematic structure of the Cs_3_BiCl_6_/GaN heterojunction photodetector, with the detailed experimental processes described in the Experimental section. According to the UV photoelectron spectroscopy (UPS) and the Tauc plot of the UV–vis absorption spectrum (Figure 2b and Figure S2a,b, Supporting Information), the valence band maximum (*E*_VBM_) and the conduction band minimum (*E*_CBM_) of Cs_3_BiCl_6_ can be ascertained through the following formulas:

(1) $$E_{\mathrm{VBM}}=hv-E_{\mathrm{Cutoff}}+E_{\mathrm{Fermi}}$$

(2) $$E_{\mathrm{CBM}}=E_{g}+E_{\mathrm{VBM}}$$

in which, *hv* is the UV radiation energy (21.22 eV), *E*_Cutoff_ is the binding energy of the secondary cutoffs (17.78 eV), *E*_Fermi_ is the difference between *E*_VBM_ and the Fermi level (3.44 eV), and *E*_g_ is the band gap energy (3.56 eV). Thus, *E*_VBM_ and *E*_CBM_ of Cs_3_BiCl_6_ are calculated as 6.88 eV and 3.32 eV, respectively. Combined with the electrical parameters of GaN known from the previous reports,^[^ ^33^ ^]^ the energy band alignment of Cs_3_BiCl_6_/GaN heterojunction is illustrated in Figure 2c. A typical type‐II heterojunction will be built once the Cs_3_BiCl_6_ films and GaN are in contact, thus a built‐in electric field will form at the hetero‐interface with the direction from GaN to Cs_3_BiCl_6_. When the device is exposed to UV light, the photogenerated electron‐hole pairs that spread over the hetero‐interface would be separated in the opposite direction by the built‐in electric field. Specifically, photogenerated holes will transport to the perovskite side while photogenerated electrons are driven toward the GaN side. As a result, the devices generate a photocurrent in the external circuit under zero bias, demonstrating self‐powered operation capability.^[^ ^34^, ^35^, ^36^ ^]^ Due to the elimination of the need for an external power supply, the self‐powered photodetectors exhibit practical advantages, such as low power consumption, simplified device architecture, and compatibility with battery‐free or remote sensing systems. Note that the built‐in electric field would impede the recombination of electrons and holes, favoring a low dark current and expecting to achieve a high specific detectivity.

To explore the spectral selectivity of the Cs_3_BiCl_6_/GaN photodetector, the response spectra of the device with different thicknesses of Cs_3_BiCl_6_ layer were measured at 0 V. As seen in Figure 2d, all the devices can respond to the 250–320 nm and 355–370 nm light bands, exhibiting self‐powered UV dual‐band photodetection feature. Moreover, as the thickness of Cs_3_BiCl_6_ increases, the left edges of both response bands move toward the long wavelength and the responsivity at 320 nm weakens. When the thickness of the Cs_3_BiCl_6_ layer reaches 550 nm, the responsivity of the device at 320 nm almost drops to 0, achieving a complete two‐band photoresponse. To quantify the spectral selectivity of the dual‐band photodetector. We fitted both response bands of the devices with varying thicknesses of the Cs_3_BiCl_6_ layer and got the full width at half‐maximum (FWHM) of both response bands. As shown in Table S1 (Supporting Information), with the increase of Cs_3_BiCl_6_ thickness from 70 to 550 nm, the FWHM of the 250–320 nm band decreases from 78.02 to 53.48 nm, while the FWHM of the 355–370 nm band narrows from 20.29 to 14.73 nm. These quantitative results demonstrate the enhanced dual‐band spectral selectivity with increasing Cs_3_BiCl_6_ thickness. In addition, to better elucidate the synergistic effects of the Cs_3_BiCl_6_/GaN interface, we measured the photoresponse spectrum of pristine GaN devices (In/GaN/Au). As shown in Figure S3 (Supporting Information), unlike the dual‐band UV response characteristics of the Cs_3_BiCl_6_/GaN heterojunction devices, the pristine GaN devices exhibit a broad photoresponse spanning from 200 to 370 nm.

To investigate the mechanisms of UV dual‐band photodetection, the absorption spectra of Cs_3_BiCl_6_ films with varying thicknesses and the GaN template were tested. Figure 2e demonstrates that all Cs_3_BiCl_6_ films exhibit two absorption bands. The absorption band at 330 nm can be attributed to the spin‐forbidden band 1S^0^−3P^1^ transition of the Bi^3+^ ions, and the doubly split peaks at the higher energy range (209 and 223 nm) can be attributed to the 1S^0^–1P^1^ transition of Bi^3+^ ion, where the split is caused by the dynamic Jahn‐Teller effect.^[^ ^26^ ^]^ The GaN template exhibits a sharp absorption edge at 370 nm. Figure 2f presents the transmission spectra of Cs_3_BiCl_6_ films with varying thicknesses and the GaN template. As the thickness of Cs_3_BiCl_6_ films increases from 70 to 550 nm, the shorter wavelength band shifts its left edge from 230 to 250 nm, while the left edge of the longer wavelength band similarly advances toward the long wavelengths. When the thickness is increased to 550 nm, the transmissivity of the light in 200–250 nm and 320–345 nm bands reaches 0, meaning that the light is completely absorbed by the Cs_3_BiCl_6_ films. But interestingly, the proposed device responds little to the light in the 200–250 nm and 320–345 nm bands and responds better to the light with a high transmissivity in the Cs_3_BiCl_6_ films, such as the 250–320 nm and 350–375 nm bands, which is due to that the undesired defect states in Cs_3_BiCl_6_ films induce strong photogenerated carrier surface recombination (SR), thus failure to contribute photocurrent.^[^ ^37^, ^38^ ^]^ The oscillatory behavior observed in the transmission and absorption spectra in Figures 2e,f originates from the interference effects. When light is reflected and transmitted at interfaces between layers with different refractive indices, the phase superposition of reflected/transmitted beams occurs. When the optical path difference (Δ) satisfies the coherence condition, constructive interference (Δ = *nλ*, *n* = 0, 1, 2…) or destructive interference (Δ = (*n* + 1/2) *λ*, *n* = 0, 1, 2…) occurs, typically manifested as periodic oscillatory peaks and valleys in the absorption or transmittance spectra. To verify the interference mechanism, the absorption and transmittance spectra of the Cs_3_BiCl_6_/quartz structure were calculated using transfer matrix simulations.^[^ ^39^, ^40^ ^]^ As shown in Figure S4 (Supporting Information), the simulated spectra align well with experimental observations, both exhibiting periodic oscillations. The narrowing oscillation period with decreasing Cs_3_BiCl_6_ thickness from 550 to 200 nm further confirms that these oscillations stem from the interference effects. By leveraging the interference phenomenon, the absorption and response spectra can be tuned by adjusting the Cs_3_BiCl_6_ thickness to meet the requirements for multifunctional optoelectronic devices.

**Figure 3:**
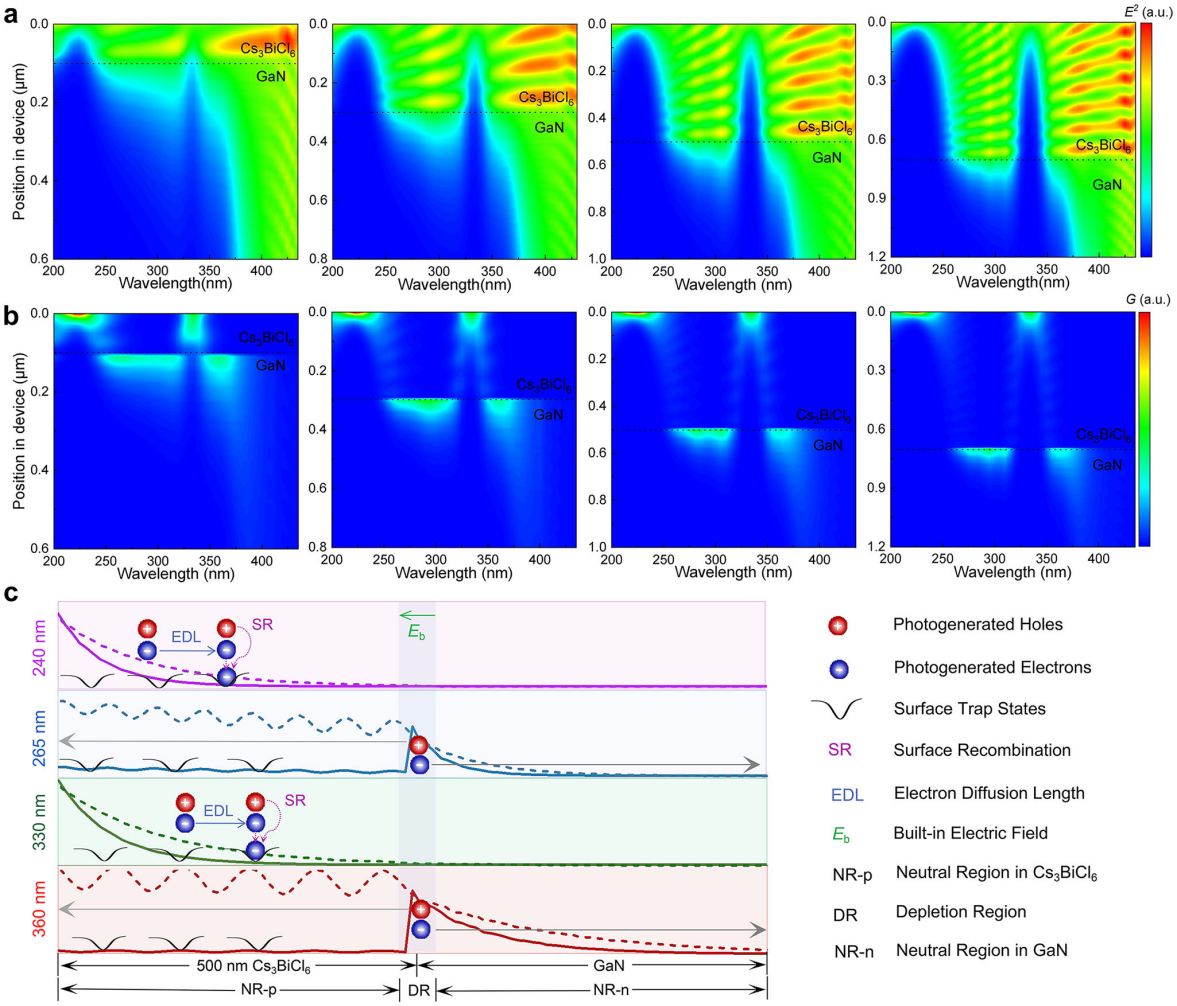
Simulated a) optical field distributions and b) photogenerated carrier distributions in devices with varying Cs_3_BiCl_6_‐layer thicknesses. c) Schematic illustration for the charge generation, surface recombination, transportation, and collection processes in Cs_3_BiCl_6_ (500 nm)/GaN heterojunction device illuminated at four representative wavelengths (240, 265, 330, and 360 nm). The gray solid line indicates the transport direction of the photogenerated carriers. The dotted curves represent the profile of the optical field distribution, and the solid curves represent the profile of the photogenerated carrier distribution.

To further investigate the working mechanisms of the proposed UV dual‐band photodetectors, the optical field and photogenerated carrier distributions in the device with varying Cs_3_BiCl_6_‐layer thicknesses (100, 300, 500, and 700 nm) were simulated based on the finite element analysis (FEA) method. The used optical constants (refractive index *n* and extinction coefficient *k*) of Cs_3_BiCl_6_ are presented in Figure S5 (Supporting Information). As shown in **Figure** **3**a, owing to the rather strong absorption of the Cs_3_BiCl_6_, the penetration depth of incident light in the 220–250 and 330–350 nm band is only 60 and 100 nm, respectively, but the light in 250–330 nm and the light with wavelengths longer than 350 nm can penetrate Cs_3_BiCl_6_ film to the interface of Cs_3_BiCl_6_/GaN heterojunction. As displayed in Figure 3b, the carriers are generated by the light within 200–250 nm and 330–350 nm bands on the surfaces of Cs_3_BiCl_6_ with a depth of <200 nm. Most carriers generated by the light within 250–330 nm and 350–375 nm bands are distributed at the Cs_3_BiCl_6_/GaN hetero‐interface. It is worth noting that the light wavelength >375 nm cannot generate photocurrent because of its smaller photon energy than the band gap of GaN.

Combined with the aforementioned findings, we thereby present the working mechanisms of the UV dual‐band heterojunction photodetector, as depicted in Figure 3c. Here, the optical field distribution and the photogenerated carrier distribution of the device illuminated at four typical wavelengths of 240, 265, 330, and 360 nm are extracted. The active layer of the photodetector can be divided into three regions: the neutral region in the Cs_3_BiCl_6_ layer (NR‐p), the depletion layer (DR), and the neutral region in the GaN layer (NR‐n). The light field distribution conforms to the Lambert‐Beer law. When the device is illuminated by 240 and 330 nm light, which is centrally located in two typical absorption bands of Cs_3_BiCl_6_, the incident light intensity decreases exponentially with the penetration depth. As a result, the photogenerated carriers are only generated in a narrow area near the surface of Cs_3_BiCl_6_. However, owing to the limited carrier diffusion length and strong surface defect state recombination aggravated by numerous grain boundaries in Cs_3_BiCl_6_ films, these photogenerated carriers can hardly arrive at the DR and thus cannot contribute to the photocurrent. When the device is illuminated by 265 and 360 nm light with low absorption coefficients for Cs_3_BiCl_6_, photons can easily penetrate Cs_3_BiCl_6_ to the DR, and photogenerated electron‐hole pairs are quickly separated by the built‐in electric field (*E*_b_), contributing to the photocurrent. In addition, the photoresponse spectra of two bands become narrower along with the thickness of Cs_3_BiCl_6_ increases (Figure 3b), consistent with the experiment results (Figure 2d), which is due to that increased thickness restricts light with short penetration depth from reaching to the DR, hence inhibiting the generation of photocurrent. Thus, the Cs_3_BiCl_6_ layer in the device can be considered as a “filter” to selectively block the light in 200–250 and 330–350 nm bands, while simultaneously combining with GaN to achieve the dual‐band photoresponse targeted at 250–330 and 350–375 nm bands. Furthermore, the photoresponse spectra of the device at different reverse bias voltages were carried out. As presented in Figure S6 (Supporting Information), applying a reverse bias with the same direction of the built‐in electric field results in a higher electric field and a broader DR, increasing the possibility of photogenerated carriers arriving at the DR and thus enhancing the responsivity. The energy band diagrams of the Cs_3_BiCl_6_/GaN heterojunction under zero and reverse bias, as presented in Figure S7 (Supporting Information), further reveal the above discussions about responsivity depending on the reverse bias.

**Figure 4:**
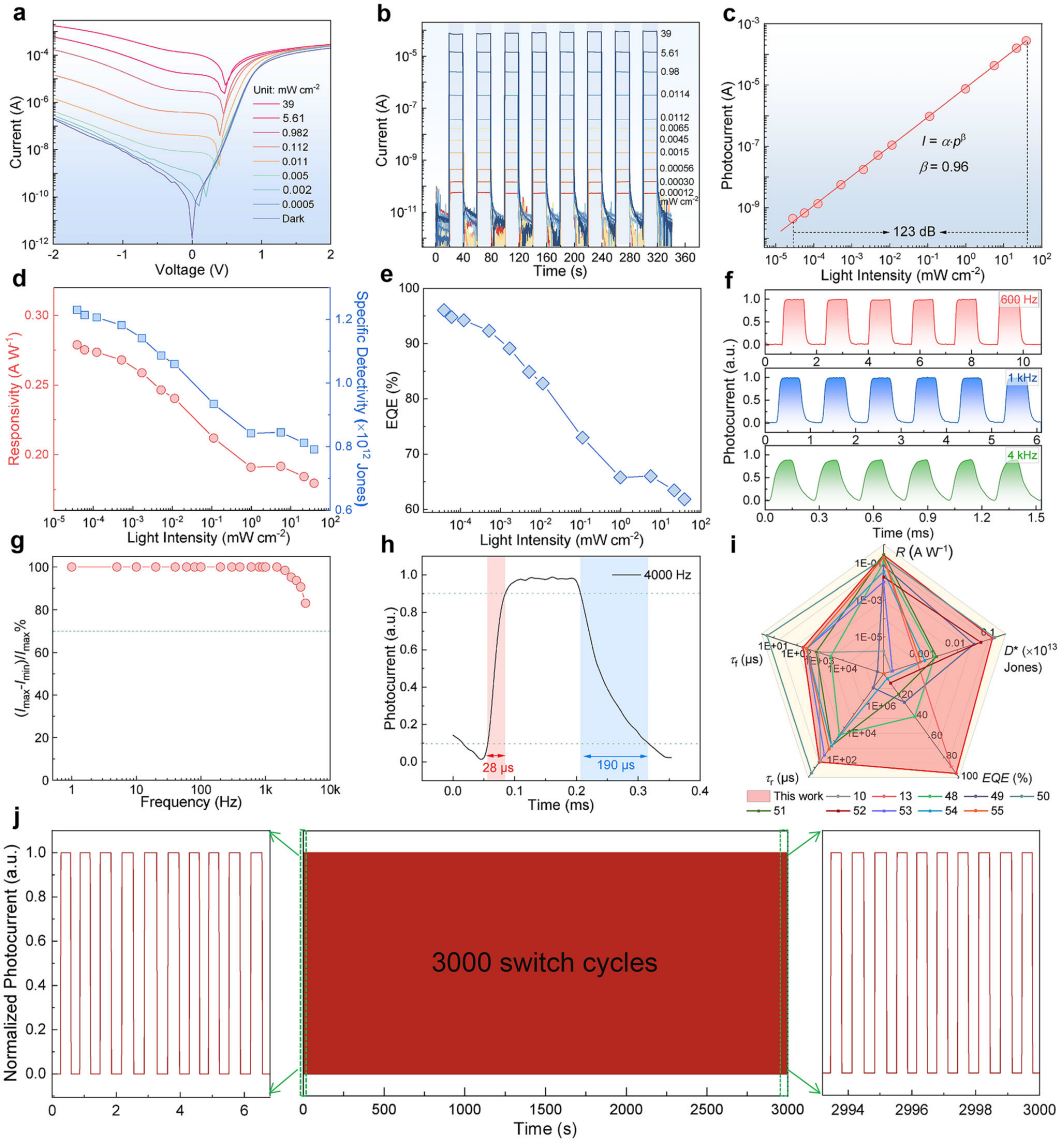
a) *I*–*V* curves of the Cs_3_BiCl_6_/GaN heterojunction photodetector measured in the dark and under various light irradiation powers (360 nm). b) *I*–*t* curves of the device tested under different light irradiation power (360 nm) at 0 V. c) Logarithmic curves of photocurrent versus light irradiation power at 0 V. d) Responsivity and specific detectivity versus light power curves. e) *EQE* versus light power curve. f) Photoresponse characteristics under pulsed light irradiation at frequencies of 600, 1000, and 4000 Hz at 0 V. g) The relative balance [(*I*_max_−*I*_min_)/*I*_max_] versus switching frequency. h) Single enlarged curve of photoresponse of the photodetector for estimating the rise/fall time. i) Comparison of key performance parameters of the as‐prepared photodetector with other dual‐band responsive photodetectors. j) Continuous 3000 response cycles stability test of the photodetector under 360 nm light irradiation at a power intensity of 1 mW cm^−2^ at 0 V.

To evaluate the photodetection performance of the Cs_3_BiCl_6_/GaN heterojunction photodetector, the incident light of 360 nm is selected as the excitation source. **Figure** **4**a shows the current‐voltage (*I*–*V*) curves of the photodetector under different light irradiation power (0–39 mW cm^−2^). One can see that, at the biases of ± 2 V, there is a large rectification ratio of > 10^3^ under the dark field. Because both Au/Cs_3_BiCl_6_ and In/GaN interfaces are Ohmic contact (Figure S8, Supporting Information), the rectification behavior was therefore considered to come from the Cs_3_BiCl_6_/GaN heterojunction. Under a light intensity of 39 mW cm^−2^, the photodetector can achieve an open‐circuit voltage (*V*_OC_) of 0.50 V and a short‐circuit current (*I*_SC_) of 0.12 mA, as shown in Figure S9 (Supporting Information), showing a remarkable photovoltaic behavior, enabling the photodetector to operate in a self‐driven mode. Figure 4b shows the current‐time (*I*–*t*) curves of the photodetector under different light irradiation power (0.12 µW cm^−2^–39 mW cm^−2^). One can see that the current reversibly switches between high and low conductance, demonstrating excellent reproducibility and stability, and generating a series of tunable on/off ratios. When the light irradiation power reaches 39 mW cm^−2^, the on–off ratio is up to 10^7^. Such a high value ensures the accuracy of the proposed photodetector to detect a weak light signal. By plotting the photocurrent (*I*_P_) values as a function of light irradiation power (*P*), a nonlinear dependence can be found. As seen in Figure 4c, by fitting the data through the power law of *I*_P_ = *α*·*P^β^*, where *α* is a constant and *β* is an exponent, a value of *β* = 0.96 is derived, close to that of an ideal photodetector (*β* = 1), suggesting a small recombination loss and the high quality of the Cs_3_BiCl_6_/GaN heterojunction. *LDR* can characterize the light power range in which the photocurrent is linearly proportional to the input light power. The *LDR* of the photodetector was calculated to be 123 dB by using the formula of *LDR* = 20 × log (*P*_sat_/*P*_low_), where *P*_sat_ and *P*_low_ are the upper and lower of light power, respectively, demonstrating that the device possesses a good linear response to the light power across a wide range. The responsivity (*R*), specific detectivity (*D* ^*^), and external quantum efficiency (*EQE*) are three key parameters to quantitatively evaluate the performance of the photodetector. *R* is determined by the following formula:

(3) $$R=\frac{I_{P}-I_{D}}{PS}$$

where *I*_P_ is the photocurrent, *I*_D_ is the dark current, and *S* is the effective area of the device. The pink dotted line in Figure 4d reflects the relationship of the responsivity with the light irradiation power at zero bias, which reaches the maximum value of 0.26 A W^−1^ at an irradiation power of 0.04 µW cm^−2^. Besides, the *D* ^*^ of the photodetector was evaluated by the following expressions:

(4) $$D^{*}=\frac{\sqrt{A\Delta f}}{NEP}$$

(5) $$NEP=\frac{\sqrt{\bar{{i_{n}}^{2}}}}{R}$$

where *A* is the effective area of the device, Δ*f* is the electrical bandwidth, *NEP* is noise equivalent power, $\sqrt{\bar{{i_{n}}^{2}}}$ is the root‐mean‐square value of the noise current. The noise (*i*_n_) of the device is determined not just by shot noise (*i*_shot_), but also by flicker 1/*f* noise (*i*_1/f_) and thermal noise (*i*_thermal_).^[^ ^41^ ^]^ To avoid the overestimation, *D* ^*^ was calculated by the following formula:

(6) $${i_{\mathrm{shot}}}^{2}=2qI_{d}\Delta f$$

(7)

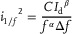

(8) $${i_{\mathrm{thermal}}}^{2}=\frac{4kT\Delta f}{R_{s}}$$

(9) $${i_{n}}^{2}={i_{\mathrm{shot}}}^{2}+{i_{1/f}}^{2}+{i_{\mathrm{thermal}}}^{2}$$

where *I*_d_ is the dark current, *f* is the frequency, and *R*_s_ is the shunt resistance. *C*, *α*, and *β* are constants. Figure S10 (Supporting Information) illustrates the relationship between $\sqrt{\bar{{i_{n}}^{2}}}$ of the device and the frequency. The measured total noise acquired from a rapid Fourier transform of the time‐domain dark current is perfectly fitted with the calculated total noise. The measured noise level per unit bandwidth was determined to be 5.67 × 10^−14^ A Hz^−1/2^, which is one order of magnitude lower than those of previously reported self‐powered perovskite/GaN heterojunction photodetectors.^[^ ^33^, ^42^ ^]^ In addition, when *f* > 100 Hz, the noise characteristics of the Cs_3_BiCl_6_/GaN heterojunction devices are predominantly governed by shot noise and thermal noise. When *f* < 100 Hz, the noise mainly originates from the 1/*f* noise. The 1/*f* noise primarily arises from the non‐uniform impurity distributions,^[^ ^43^ ^]^ microcracks in the photosensitive layer,^[^ ^44^ ^]^ or scattering at the interface between different functional layers.^[^ ^45^ ^]^ The as‐prepared Cs_3_BiCl_6_ films demonstrate crack‐free morphology with densely packed crystalline grains, effectively suppressing 1/*f* noise generation. Consequently, the *D* ^*^ of the device was calculated. As seen in Figure 4d, the *D* ^*^ value decreases gradually with increasing light irradiation power, and the maximum value was calculated to be 1.23 × 10^12^ Jones (1 Jones = 1 cm Hz^1/2^ W^−1^) at the exciting light power of 0.04 µW cm^−2^, implying an outstanding weak‐light detection capability. In addition, the *EQE* of the photodetector can be ascertained by the following expression:

(10) $$EQE=\frac{Rhc}{e\lambda }$$

in which *λ* is the exciting light wavelength, *h* is Planck's constant, *c* is the light speed, and *e* is the elementary charge. As presented in Figure 4e, the highest *EQE* is 96% at the exciting light power of 0.04 µW cm^−2^. One can observe that *R*, *D* ^*^, and *EQE* values display a decreasing trend with light irradiation power, which can be related to the photocurrent saturation caused by the increased carrier recombination probability at higher light irradiation.

The response speed is another significant parameter of photodetectors to assess their ability to track rapidly switched optical signals. Figure 4f presents the normalized photoresponse of the device to pulsed light with frequencies of 0.6, 1, and 4 kHz, from which we can observe that the photodetector can operate with outstanding repeatability in a wide frequency range. The relative balance [(*I*_max_–*I*_min_)/*I*_max_] depending on switching frequency is shown in Figure 4g, and the 3dB frequency of >4 kHz suggests that the Cs_3_BiCl_6_/GaN heterojunction photodetector can track ultrafast optical signals. According to the photocurrent response characteristics captured at 4 kHz, we further estimate the rise/fall time (*τ*_r_/*τ*_f_) of the device. As seen in Figure 4h, the *τ*_r_/*τ*_f_ was determined as 28/190 µs, suggesting that the proposed heterojunction photodetector has the ability to follow a fast varying light signal. It should be noted that the device exhibits a noticeable difference between rise time and fall time. At the moment of transition from darkness to light, photogenerated carriers are generated within femtosecond to picosecond timescales.^[^ ^46^ ^]^ The built‐in electric field enables prompt separation and collection of photogenerated carriers, leading to a short *τ*_r_. During the light‐to‐dark transition, the fall time is dominated by carrier recombination. Although direct electron‐hole recombination can occur within a picosecond range,^[^ ^46^ ^]^ defects in Cs_3_BiCl_6_ and GaN may prolong the recombination time to the millisecond scale due to the trap‐assisted de‐trapping processes at the heterointerface or within the bulk materials.^[^ ^47^ ^]^ In addition, the nonequilibrium transport of electrons and holes causes the majority of carriers to slowly migrate to the external circuit, thus leading to a long *τ*_f_.^[^ ^47^ ^]^

Figure 4i and Table S2 (Supporting Information) present the comparison of key performance parameters and response band of the as‐prepared photodetector with other reported dual‐band photodetectors, from which we can observe the proposed device based on Cs_3_BiCl_6_/GaN heterojunction not only exhibits unique self‐powered dual‐band UV detection capabilities but also achieves state‐of‐the‐art performance in *R*, D*, and *EQE* compared to existing counterparts.^[^ ^10^, ^13^, ^48^, ^49^, ^50^, ^51^, ^52^, ^53^, ^54^, ^55^ ^]^ As is well known, the working stability of perovskite‐based optoelectronic devices has always been a challenging issue. To further examine the long‐term stability of the unencapsulated photodetector, temporal photocurrent response with 3000 switch cycles was tested in air ambient (25 °C, 30–45% humidity), where the light excitation intensity and a zero bias were fixed. As shown in Figure 4j, the device can switch reversibly between the on and off states, and both the photocurrent and dark current maintain their original values after 3000 cycles of operation. In addition, we have conducted continuous illumination (360 nm, 30 µW cm^−2^) stability tests. As shown in Figure S11 (Supporting Information), the device maintains ≈91% of the initial photocurrent after continuous illumination for 20 000 s. The results indicate that the proposed photodetector has excellent long‐term working stability, and suggest that the lead‐free Cs_3_BiCl_6_ can serve as a reliable light absorber for photodetection applications.

**Figure 5:**
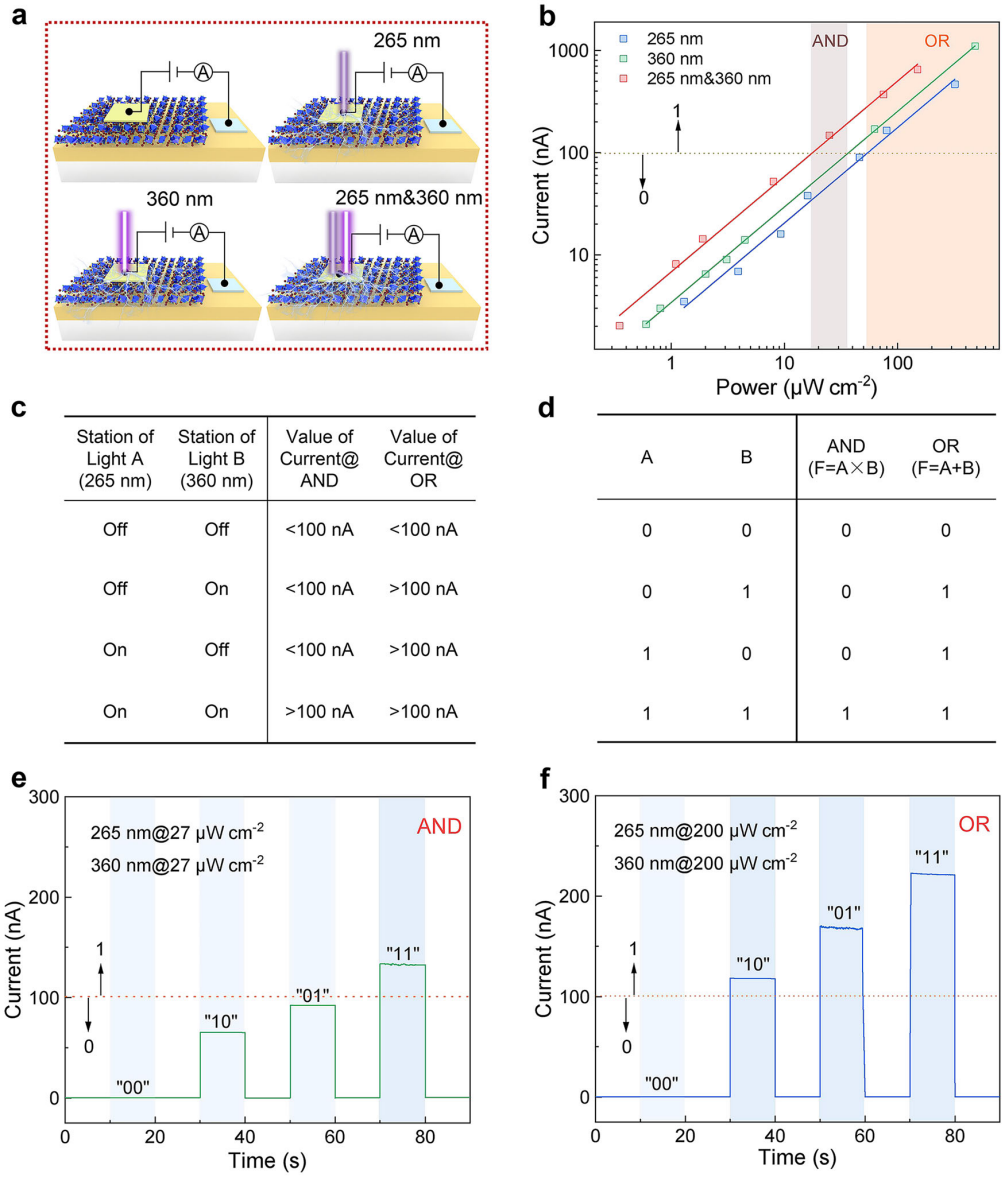
a) Schematic diagram of four optical signal inputs. b) Photocurrent versus light irradiation power curves of the photodetector under 265 nm, 360 nm, and mixed light (265 and 360 nm) irradiation at 0 V. c) Condition table and d) truth table of “AND” and “OR” gates. *I*–*t* curves of e) “AND” and f) “OR” gates.

Optoelectronic logic gates have received explosive attention as pivotal blocks of the prospective digital computer for processing information accurately and quickly.^[^ ^8^, ^56^, ^57^ ^]^ Given the self‐powered ultrafast dual‐band photoresponse characteristics of the Cs_3_BiCl_6_/GaN heterojunction photodetector, it is expected to implement the “AND” and “OR” gates with low power consumption and fast calculating ability. As illustrated in **Figure** **5**a, four optical signal inputs are regulated by controlling the switch station of 265 nm and 360 nm light. To manufacture the “AND” and “OR” gates, we determined firstly the photocurrent versus light irradiation power curves of the device excited by only 265 nm, only 360 nm, and the mixed light (265 nm & 360 nm), separately. As presented in Figure 5b, the photocurrent increases linearly with increasing light irradiation power under three conditions. When the incident light power is in the range of 18–36 µW cm^−2^, the output photocurrent generated by inputting only 265 nm or 360 nm light is <100 nA; but when both UV lights were provided simultaneously, the photocurrent is over 100 nA. When the incident light power exceeds 52 µW cm^−2^, the monochromatic and mixed light (265 and 360 nm) yield photocurrents above 100 nA. As a result, the switch station and light power of 265 nm and 360 nm can be considered as the optical gate modulators, and are expected to achieve a multi‐state logic gate controlled by light. Figure 5c presents the condition table of “AND” and “OR” logical circuits in this logic system. The condition of light can be either “on” or “off”, and the condition of photocurrent can be either “>100 nA” or “<100 nA”. If the light being turned on is represented by “1”, the light being turned off is represented by “0”, the output current above 100 nA (the yellow dashed line in Figure 5b delegates the fiducial level) is set to “1”, and the output current below 100 nA is set to “0”, thus the condition table can be converted to the truth table of “AND” and “OR” logical circuits (Figure 5d). As presented in Figures 5e,f, the device will implement the “AND” gate when the incident light power is in the range of 18–36 µW cm^−2^, and execute the “OR” gate when the incident light power exceeds 52 µW cm^−2^. Thus, the “AND” and “OR” gates with no power consumption are successfully achieved in one Cs_3_BiCl_6_/GaN heterojunction photodetector by controlling the switch station and irradiation power of incident light.

**Figure 6:**
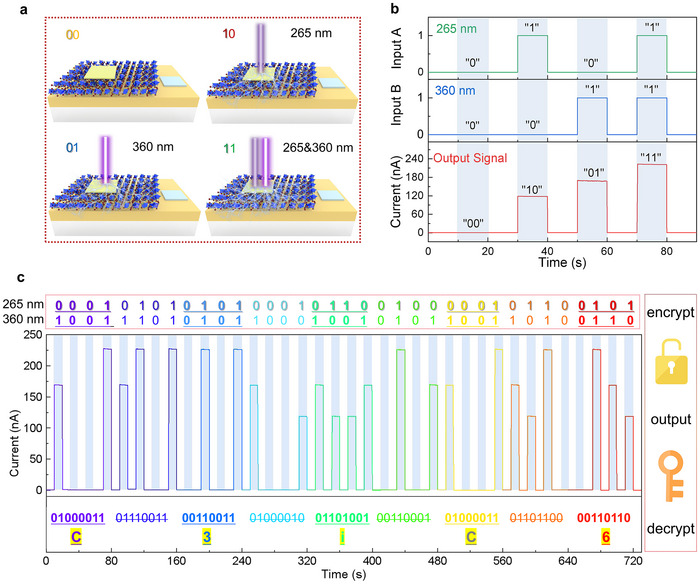
a) Schematic diagram of four light input signals corresponding to different light irradiation. b) Reprogrammable electrical output signals corresponding to different light input signals. The light power of both 265 nm and 365 nm is 200 µW cm^−2^. c) Schematic diagram of encrypted photo‐communication. Here, the “C3iC6” is decrypted according to the ASCII codes and the agreed effective signal segments (the effective digital data is underlined).

The realization of encrypted photo‐communication technology is of great significance to the development of informatization in 5G, the Internet of Things, and cloud computing. The encrypted photo‐communication depends on the data encoding process through the modulation of optical signals and the decoding process of the electrical signal converted by the photodetectors. The unique UV dual‐band photoresponse characteristic of Cs_3_BiCl_6_/GaN heterojunction photodetector endows the device with tremendous potential for encrypted photo‐communication applications. As illustrated in **Figure** **6**a, we used the UV‐B light (360 nm) signals with the adjunct of the not easily captured solar‐blind light (265 nm) signals to transmit digital data of “00”, “10”, “01”, and “11”. As displayed in Figure 6b, when the device is irradiated without a light source (“00”), monochromatic solar‐blind light (“10”), monochromatic UV‐B light (“01”), and mixed light (“11”), the output current is ≈0, 118, 168, and 221 nA, respectively. As shown in Figure 6c, first, the encryption of the information was executed by assembling diverse digital data and introducing periodic effective signal segments (the effective digital data is underlined). Then, the Cs_3_BiCl_6_/GaN photodetector will respond to the optical signal and output homologous photocurrent arrangements. Finally, according to the ASCII code and agreed effective signal segments, the correct information of “C3iC6” was decrypted. Notably, when this double‐encrypted transmission signal is intercepted, the information would be incorrectly decrypted if the photodetector can only recognize the monochromatic UV‐B light signal or the eavesdropper is unaware of the agreed effective signal segment. Therefore, the UV dual‐band response characteristic makes the Cs_3_BiCl_6_/GaN heterojunction photodetector have great potential in encrypted photo‐communication and provides an opportunity for advanced encryption technology compatible with practical applications.

## 3 Conclusion

In summary, we have successfully fabricated the self‐powered UV dual‐band photodetectors based on Cs_3_BiCl_6_/GaN heterojunction through a dual‐source co‐evaporation technique. The experimental results and theoretical simulation confirm that the characteristic UV dual‐band photoresponse originates from the special two‐band absorption of Cs_3_BiCl_6_ and its strong surface‐charge recombination behavior. Because of the high material integrity of Cs_3_BiCl_6_ and efficient interfacial charge transfer, the proposed photodetectors demonstrate excellent performances including ultra‐high *I*_on_/*I*_off_ ratio of 1 × 10^7^, large specific detectivity of 1.23 × 10^12^ Jones, and ultrafast response speed of τ_r_/τ_f_ = 28 µs/190 µs. Moreover, the unencapsulated device exhibits good long‐term working stability. Finally, using self‐powered UV dual‐band characteristics of the device, “AND” and “OR” optoelectronic logic gates and encrypted photo‐communication were successfully realized. It is believed that this work provides new insights into the manufacture of high‐performance self‐powered dual‐band photodetectors to serve the modern optoelectronic interconnected circuits.

## 4 Experimental Section

### Materials

Cesium chloride (CsCl, 99.99%) and bismuth chloride (BiCl_3_, 99.99%) were purchased from Xi'an Polymer Light Technology Corp. All these chemical materials were directly used without further purification.

### Preparation of Cs_3_BiCl_6_ Films

Before deposition, a commercially available GaN template was cleaned ultrasonically with acetone, ethanol, and deionized water for 10 min, respectively, and was disposed of by Ar gas plasma for 15 min to improve the wettability. Here, the Cs_3_BiCl_6_ films were deposited via the dual‐source co‐evaporation technique. First, the mixture of CsCl and BiCl_3_ powders in a molar ratio of 3:1 was fully ground for 30 min. Then, the powders were transferred to a tungsten boat, and the substrate was loaded into the rotating basalia in the evaporation chamber whose base pressure was lower than 9.9 × 10^−4^ Pa. The basalia was maintained at 150 °C, and the evaporation rate was kept at 1 Å/s. The Cs_3_BiCl_6_ films with different thicknesses were prepared by changing the molar quantity of CsCl and BiCl_3_. After the evaporation, the Cs_3_BiCl_6_ films were transferred to a hot plate with temperatures of 150 °C for 1 h to improve the quality of the films.

### Fabrication of the Devices

An In electrode (80 nm) on GaN and an Au electrode (50 nm) on the Cs_3_BiCl_6_ films were fabricated by e‐beam evaporation using a shadow mask. The active area of the device was 0.25 mm^2^.

### Characterization of Materials and Devices

The morphology of the prepared Cs_3_BiCl_6_ films was examined through field‐emission SEM (Jeol‐7500F, 15 keV). The composition of the element in the Cs_3_BiCl_6_ films was analyzed through the EDS attached to the SEM. The crystallinity of Cs_3_BiCl_6_ films was measured by XRD (Panalytical X'Pert Pro). The chemical composition and valence states of the Cs_3_BiCl_6_ films were performed by XPS (SPECS XR50). The absorption and transmission spectra were tested through a UV–vis spectrophotometer (Unico, UV‐4802). The optical constants (*n* and *k*) of the Cs_3_BiCl_6_ films were tested by a spectroscopic ellipsometer (Eoptics, SE‐VE). The photoelectrical performance of the device was measured via a testing system including light sources, a grating spectrograph (Shanghai PRECISION Instruments Co., Ltd, Omni‐λ300), a multi‐channel electrical test system (Keithley, 2636B), an optical chopper (SRS, SR540), and an oscilloscope (Tektronix, DPO2012B) in air.

### Optical Simulation

The optical field distribution in the device was simulated using the wave optics module in the COMSOL simulation tool. The optical constants of all the layers of the device were used to run the simulation. Optical constants of GaN, In, and Au materials could be found in published results and an online database.^[^ ^58^, ^59^ ^]^ The electromagnetic field distribution could be attained by solving Maxwell's equation, which allowed us to obtain the distribution of photogenerated carriers *G* by using the following equation

(11) $$G=real\left|\nabla \cdot P_{\mathrm{op}}\right|\times \frac{\eta }{h\lambda }$$

where *η* was the quantum yield assumed to be equal to the unity, *P*_op_ was a time‐varying power flow under the light field, *h* was the Planck constant, and *λ* was the wavelength of incident light.

## Conflict of Interest

The authors declare no conflict of interest.

## Supporting information



Supporting Information

## Data Availability

The data that support the findings of this study are available from the corresponding author upon reasonable request.
