# Supplementary material for: Self‐Powered UV Dual‐Band Photodetector Based on Cs3BiCl6/GaN Heterojunction for Logical Operation and Encrypted Photo‐Communication
Source: Adv Sci (Weinh). 2025 May 11;12(28):2503498. doi: 10.1002/advs.202503498 (PMC12302572; doi:10.1002/advs.202503498)
Supplement: Supplementary file 1 — Supporting Information [file ADVS-12-2503498-s001.docx]

Copyright WILEY-VCH Verlag GmbH & Co. KGaA, 69469 Weinheim, Germany, 2025.

Supporting Information

**Self-Powered Ultraviolet Dual-Band Photodetector Based on Cs_3_BiCl_6_/GaN Heterojunction for Logical Operation and Encrypted Photo-Communication**

*Jingli Ma*, *Fei Zhang, Yakun Xing, Huifang Jiang, Yongtao Tian, Huifang Ji, Xu Chen, Di Wu, Longhui Zeng, Xinjian Li, Chongxin Shan, and Zhifeng Shi**

Dr. Jingli Ma, Dr. Yakun Xing, Dr. Huifang Jiang, Dr. Yongtao Tian, Dr. Huifang Ji, Prof. Di Wu, Prof. Longhui Zeng, Prof. Xinjian Li, Prof. Chongxin Shan, Prof. Zhifeng Shi

Key Laboratory of Materials Physics of Ministry of Education, School of Physics, Zhengzhou University, Daxue Road 75, Zhengzhou 450052, China

E-mail: shizf@zzu.edu.cn

Dr. Fei Zhang

School of Flexible Electronics (SoFE), Henan Institute of Flexible Electronics (HIFE), Henan University, 379 Mingli Road, Zhengzhou 450046, China

Dr. Xu Chen

Department of Applied Physics, KTH Royal Institute of Technology, S-10691 Stockholm, Sweden

Keywords: ultraviolet dual-band photodetector, lead-free perovskite, Cs_3_BiCl_6_, logical operation, encrypted photo-communication


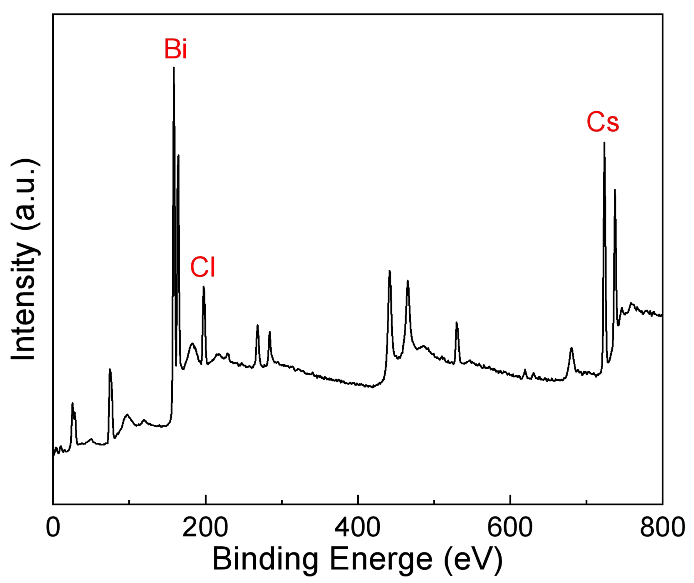


**Figure S1.** Total XPS spectrum of the as-prepared Cs_3_BiCl_6_ films.


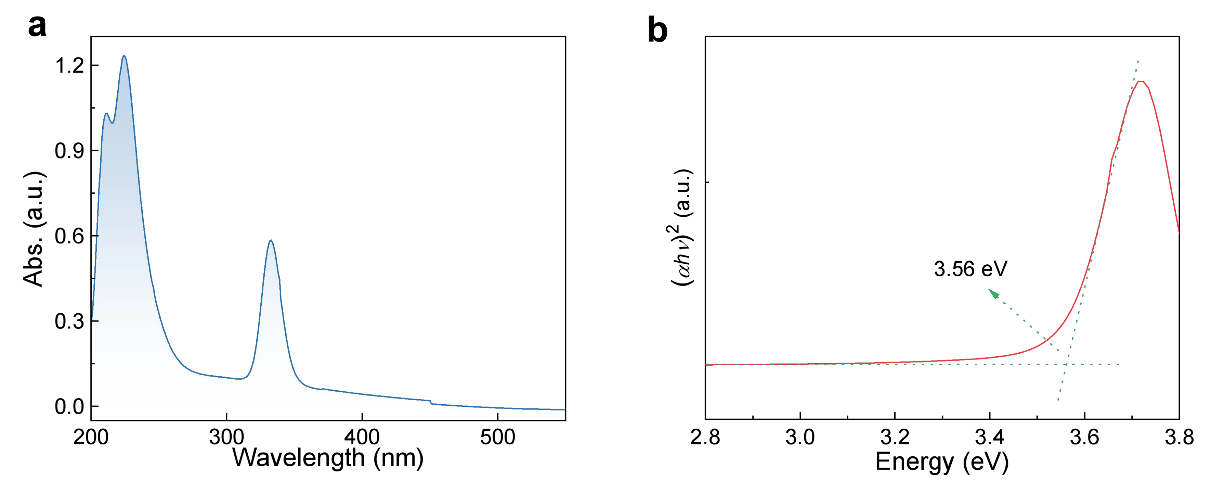


**Figure S2.** a) Absorption spectra and b) the corresponding Tauc curve of Cs_3_BiCl_6_ films. The band gap energy (*E*_g_) is 3.56 eV.


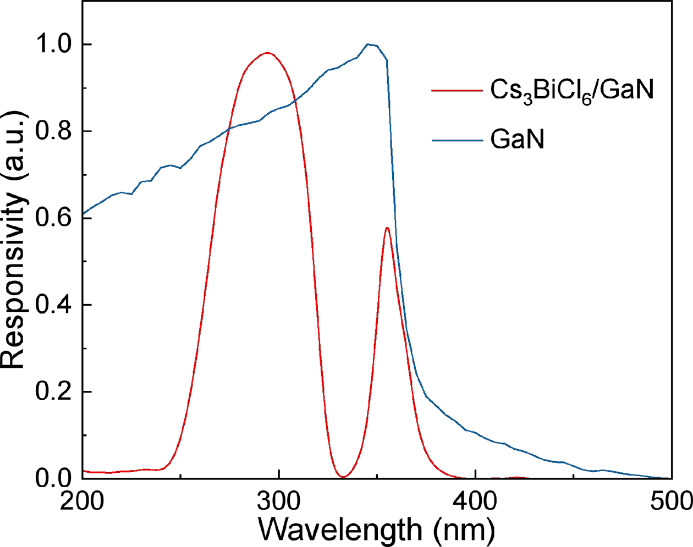


**Figure S3**. Normalized response spectra of the pristine GaN devices and the Cs_3_BiCl_6_/GaN heterojunction devices with 550 nm Cs_3_BiCl_6_ layer at 0 V.


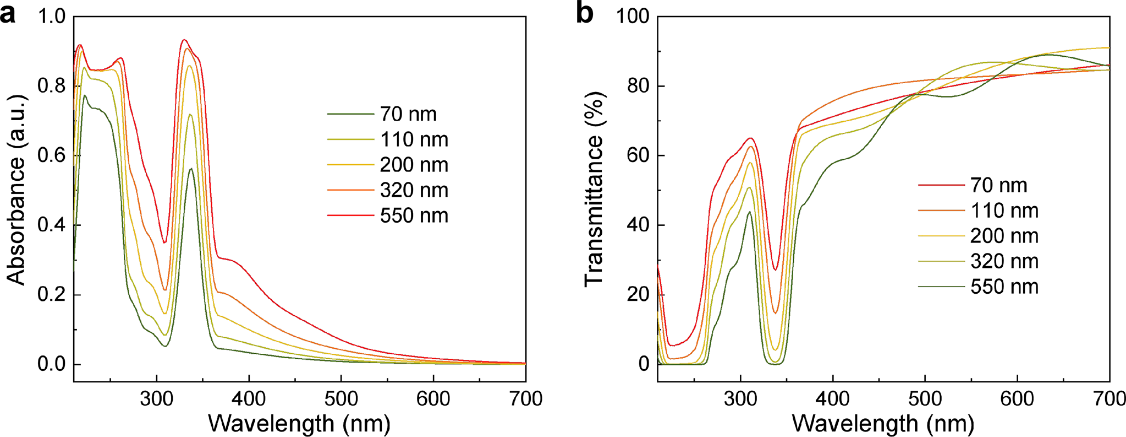


**Figure S4**. Simulated a) absorption spectra and b) transmission spectra of the Cs_3_BiCl_6_/quartz structure with varying thicknesses Cs_3_BiCl_6_.


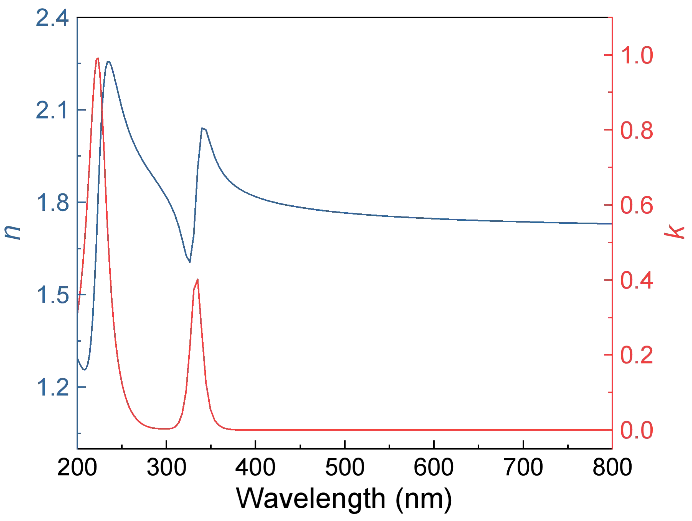


**Figure S5.** Extinction coefficient *k* and refractive index *n* of the as-prepared Cs_3_BiCl_6_ films.


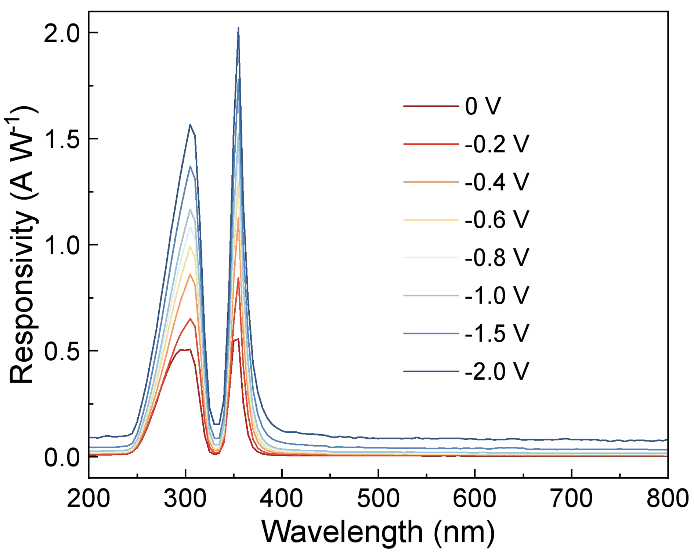


**Figure S6.** Response spectra of the photodetector under different bias voltages.


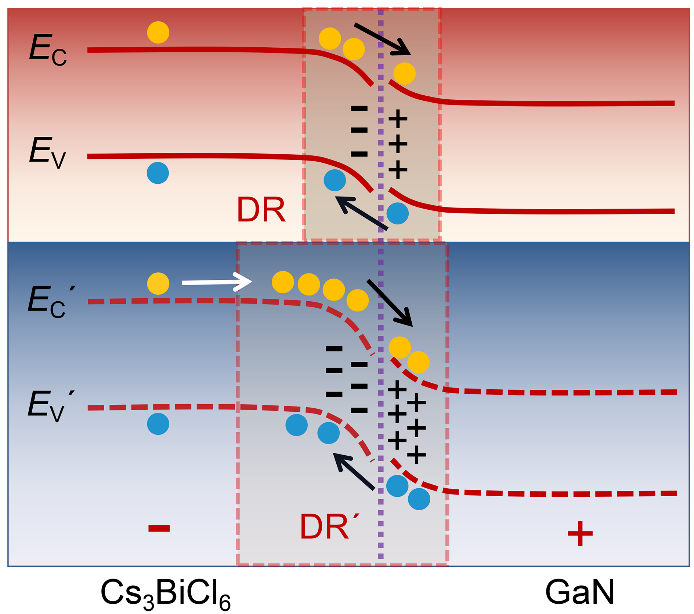


**Figure S7.** Energy band schematics of the Cs_3_BiCl_6_/GaN heterojunction under zero and reverse bias.


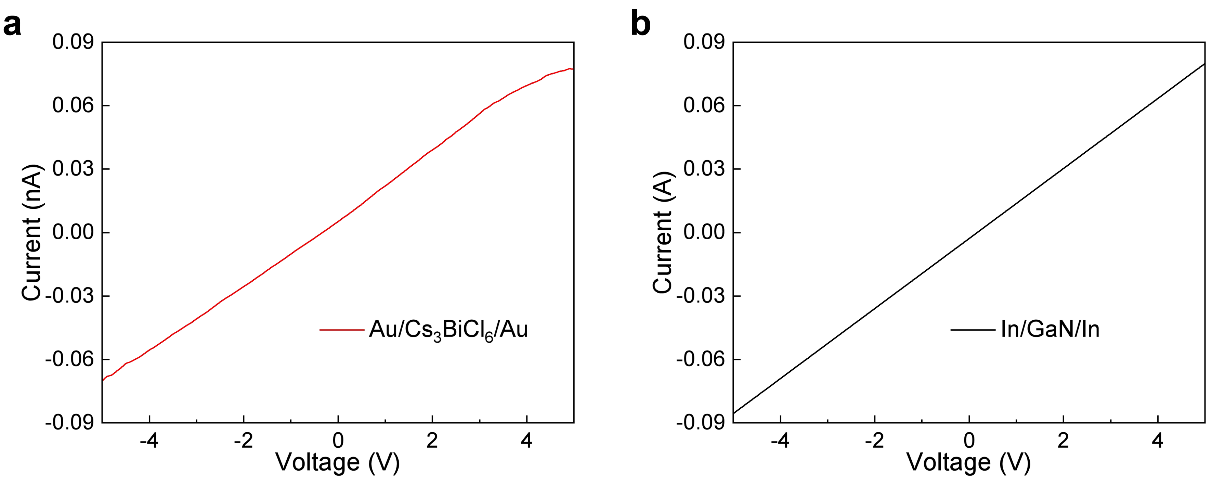


**Figure S8.** Current−voltage curves of a) Au/Cs_3_BiCl_6_/Au device and b) In/GaN/In device. Both the Au/Cs_3_BiCl_6_ and In/GaN interfaces are confirmed to be in Ohmic contact *via* the linear *I*−*V* relationships of the Au/Cs_3_BiCl_6_/Au and In/GaN/In structures.


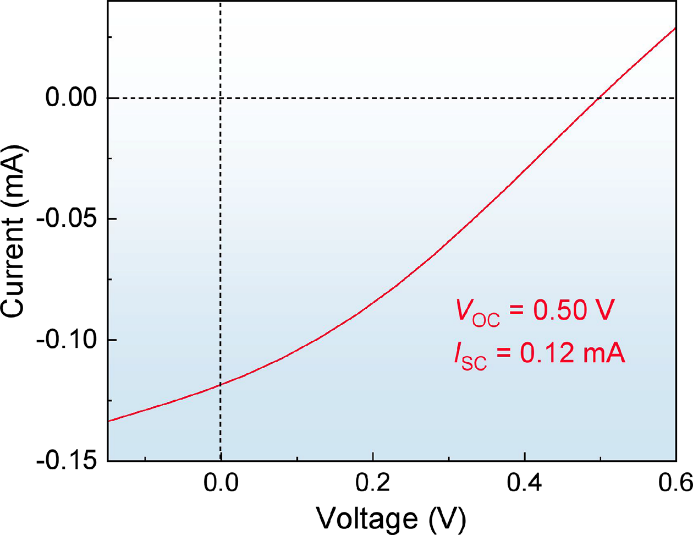


**Figure S9.** Enlarged *I*−*V* curve showing the photovoltaic effect of the photodetector.


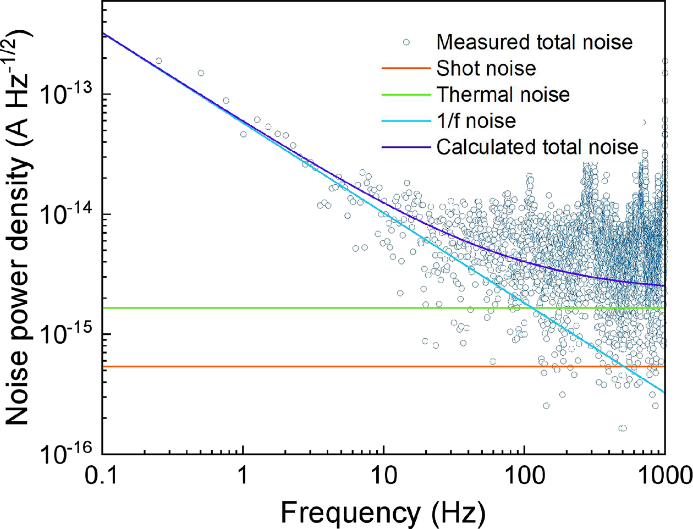


**Figure S10**. Noise current of the photodetector as a function of frequency.


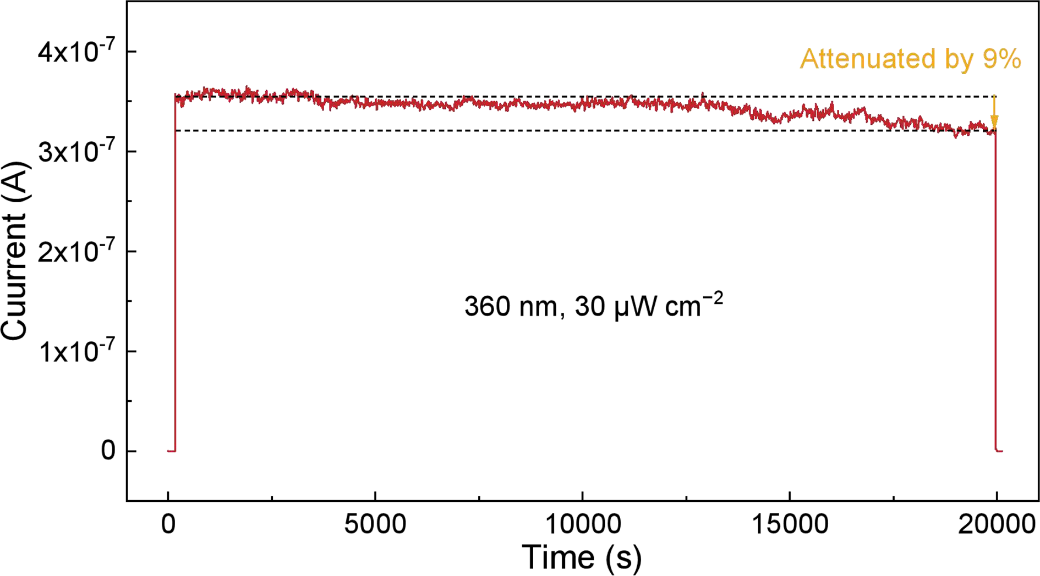


**Figure S11**. Continuous illumination stability test of the photodetector under 360 nm light irradiation at a power intensity of 30 μW cm^−2^ at 0 V.

**Table S1**. FWHM of two response bands of the photodetectors with varying thicknesses of Cs_3_BiCl_6_ layer.

| Thicknesses | FWHM of 250–320 nm band | FWHM of 355–370 nm band |
| --- | --- | --- |
| 70 nm | 78.02 nm | 20.29 nm |
| 110 nm | 70.31 nm | 19.54 nm |
| 200 nm | 63.30 nm | 15.43 nm |
| 320 nm | 62.25 nm | 14.97 nm |
| 550 nm | 53.48 nm | 14.73 nm |

**Table S2**. Comparison of key performance metrics and response band of the as-prepared photodetector with other reported dual-band photodetectors. *λ*_ex_ is the excitation wavelength.

| Device structure | Response band (nm) | Bias (V)/*λ*_ex_ (nm) | *R*  (A W^−1^) | *D**  (Jones) | *EQE*  (%) | *τ*_r_/*τ*_f_  (ms/ms) | Ref. |
| --- | --- | --- | --- | --- | --- | --- | --- |
| Cs_3_BiCl_6_/GaN | 250–320/  355–370 | 0/360 | 0.275 | 1.23 × 10^12^ | 96 | 0.028/  0.190 | This work |
| BeZnO | 230−280/  280−400 | 0/330 | 1.4 × 10^−6^ | 1.4 × 10^9^ | − | 0.035/  0.880 | [1] |
| Cs_2_AgBiBr_6_/  SnO_2_ | 320−400/  420−520 | 0/350 | 0.11 | 2.1 × 10^10^ | 40 | 3/3 | [2] |
| CuI/CsCu_2_I_3_/GaN | 300−400/  650−730 | 0/340 | 0.071 | 3.3 × 10^11^ | 26 | 8800/  320 | [3] |
| Cs_2_SnI_6_/ZnO | 280−385/  600−900 | 15/378 | 0.183 | 1.39 × 10^12^ | 60 | 0.0043/  0.0052 | [4] |
| Al_0.5_Ga_0.5_N/  AlN | 210−256/  256−290 | 100/266 | 0.19 | − | 89 | − | [5] |
| Si/PbS | 500−1200/  1900−2200 | 0.15/  2000 | 0.29 | 2.4 × 10^10^ | 18 | 0.47/0.71 | [6] |
| (Al_0.4_Ga_0.6_)_2_O_3_/Al_0.32_Ga_0.68_N | 230−270/  280−320 | 10/305 | 0.017 | 5.19 × 10^11^ | 7 | − | [7] |
| Cs_2_AgBiBr_6_ | 500−570/  570−650 | 5/530 | 0.0096 | 1.1 × 10^9^ | − | 92/271 | [8] |
| MAPbCl_3_/  Bi-MAPbBr_3_/Bi-MAPbI_2.5_Br_0.5_/MAPbI_3_ | 365-420/  750-860 | 30/365 | 0.035 | 1.05 × 10^10^ | 3 | 0.46/0.28 | [9] |
| ZnO/CDT-TQ:PC_71_BM/  PEDOT:PSS/P3HT:PC_71_BM/ZnO | 400-700/  700-1400 | 0.5/600 | 0.2 | 7.8 × 10^9^ | − | 0.27/0.27 | [10] |

**References**

1. L. Su, H. Chen, X. Xu, X. Fang, Novel BeZnO Based Self-Powered Dual-Color UV Photodetector Realized Via a One-Step Fabrication Method. *Laser Photon. Rev.* **2017**, *11*, 1700222.
2. C. Wu, B. Du, W. Luo, Y. Liu, T. Li, D. Wang, X. Guo, H. Ting, Z. Fang, S. Wang, Z. Chen, Y. Chen, L. Xiao, Highly Efficient and Stable Self-Powered Ultraviolet and Deep-Blue Photodetector Based on Cs_2_AgBiBr_6_/SnO_2_ Heterojunction. *Adv. Opt. Mater.* **2018**, *6*, 1800811.
3. X. Zhou, C. Wang, J. Luo, L. Zhang, F. Zhao, Q. Ke, High-Performance Self-Powered UV/Red Dual-Wavelength Photodetector Based on CuI/CsCu_2_I_3_/GaN Heterojunction. *Chem. Eng. J.* **2022**, *450*, 136364.
4. D. Shao, W. Zhu, G. Xin, X. Liu, T. Wang, S. Shi, J. Lian, S. Sawyer, A High Performance UV–Visible Dual-Band Photodetector Based on an Inorganic Cs_2_SnI_6_ Perovskite/ZnO Heterojunction Structure. *J. Mater. Chem. C* **2020**, *8*, 1819.
5. Y. Chen, X. Zhou, Z. Zhang, G. Miao, H. Jiang, Z. Li, H. Song, Dual-Band Solar-Blind UV Photodetectors Based on AlGaN/AlN Superlattices. *Mater. Lett.* **2021**, *291*, 129583.
6. C. h. Xu, S. H. Luo, Y. Wang, X. F. Shi, C. Fu, J. Wang, C. Y. Wu, L. B. Luo, Bias‐Selectable Si Nanowires/PbS Nanocrystalline Film N–N Heterojunction for NIR/SWIR Dual‐Band Photodetection. *Adv. Funct. Mater.* **2023,** *33*, 2214996.
7. S. Wu, Z. Wu, P. Tian, J. Hoo, S. Guo, Z. Fang, High-Performance (Al_0.4_Ga_0.6_)_2_O_3_/Al_0.32_Ga_0.68_N-Based UVC/UVB Tunable Dual-Band Photodetectors. *CrystEngComm* **2024**, *26*, 2551.
8. S. R. Sridhar, N. K. Tailor, S. Satapathi, B. Kumar, Narrow Dual-Band Photodetector Based on Cs_2_AgBiBr_6_ Lead-Free Double Perovskite Single Crystal. *IEEE Trans. Electron Dev.* **2024**, *71*, 6838.
9. Y. Pan, X. Wang, D. Zhang, Z. Wei, Y. Xu, Y. Li, Q. Li, Z. Zhao, Z. Zhu, B. S. Bae, D. C. Onwudiwe, X. Xu, W. Lei, Visible Elimination, Ultraviolet and near-Infrared Dual-Band Photodetector Based on Single-Crystal Perovskite Heterojunctions toward Secure Optical Communication. *ACS Photonics* **2024**, *11*, 1252.
10. N. Li, N. Eedugurala, J. D. Azoulay, T. N. Ng, A Filterless Organic Photodetector Electrically Switchable between Visible and Infrared Detection. *Cell Reports Physical Science* **2022**, *3*. 100711.
